# Poly[(μ_4_-benzene-1,3,5-tricarboxyl­ato)bis­(dimethyl sulfoxide-κ*O*)­neodymium(III)]

**DOI:** 10.1107/S1600536811049622

**Published:** 2011-11-25

**Authors:** Zhongyue Li, Kun Liu

**Affiliations:** a The Department of Physics–Chemistry, Henan Polytechnic University, Jiaozuo 454000, People’s Republic of China

## Abstract

The asymmetric unit of the title compound, [Nd(C_9_H_3_O_6_)(C_2_H_6_OS)_2_]_*n*_, contains one Nd^3+^ ion, one benzene-1,3,5-tricarb­oxy­lic ligand and two coordinating dimethyl sulfoxide mol­ecules. The Nd^3+^ ion is coordinated by six O atoms from four carboxyl­ate groups of the benzene-1,3,5-tricarboxyl­ate ligands and two O atoms from two dimethyl sulfoxide mol­ecules. The metal-organic cluster formed upon symmetry expansion of the asymmetric unit consists of two metal atoms and four benzene-1,3,5-tricarboxyl­ate groups, creating a paddle-wheel-type building block arrangement. The remaining coordination sites are occupied by additional benzene-1,3,5-tricarboxyl­ate groups and dimethyl sulfoxide mol­ecules, forming a three-dimensional polymeric rare earth metal-organic framework structure.

## Related literature

For metal-organic framework structures with adsorption, catalytic and fluorescence properties, see: Sun *et al.* (2006 ▶); Ravon *et al.* (2008 ▶); Allendorf *et al.* (2009 ▶). For isostructural rare earth complexes, see: Thirumurugan & Natarajan (2004 ▶); For rare earth coordination polymers, see: Guo *et al.* (2006 ▶).
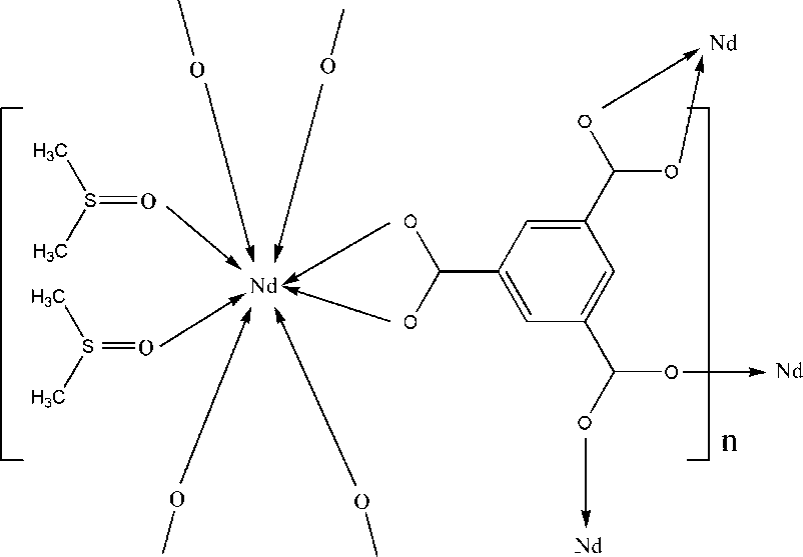


## Experimental

### 

#### Crystal data

[Nd(C_9_H_3_O_6_)(C_2_H_6_OS)_2_]
*M* *_r_* = 507.61
Monoclinic, 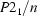

*a* = 10.380 (2) Å
*b* = 10.752 (3) Å
*c* = 16.025 (4) Å
β = 106.419 (4)°
*V* = 1715.6 (7) Å^3^
*Z* = 4
Mo *K*α radiation
μ = 3.31 mm^−1^
*T* = 273 K
0.50 × 0.40 × 0.40 mm

#### Data collection

Bruker APEX CCD diffractometer
Absorption correction: multi-scan (*SADABS*; Sheldrick, 2008 ▶) *T*_min_ = 0.289, *T*_max_ = 0.351
8925 measured reflections
3008 independent reflections
2453 reflections with *I* > 2σ(*I*)
*R*_int_ = 0.049

#### Refinement

*R*[*F* ^2^ > 2σ(*F* ^2^)] = 0.039
*wR*(*F* ^2^) = 0.082
*S* = 0.98
3008 reflections
221 parameters
H-atom parameters constrained
Δρ_max_ = 1.92 e Å^−3^
Δρ_min_ = −0.83 e Å^−3^

### 

Data collection: *SMART* (Bruker, 2001 ▶); cell refinement: *SAINT* (Bruker, 2001 ▶); data reduction: *SAINT*; program(s) used to solve structure: *SHELXS97* (Sheldrick, 2008 ▶); program(s) used to refine structure: *SHELXL97* (Sheldrick, 2008 ▶); molecular graphics: *SHELXTL* (Sheldrick, 2008 ▶); software used to prepare material for publication: *SHELXTL*.

## Supplementary Material

Crystal structure: contains datablock(s) I, global. DOI: 10.1107/S1600536811049622/jj2108sup1.cif

Structure factors: contains datablock(s) I. DOI: 10.1107/S1600536811049622/jj2108Isup2.hkl

Additional supplementary materials:  crystallographic information; 3D view; checkCIF report

## Figures and Tables

**Fig. 1.**
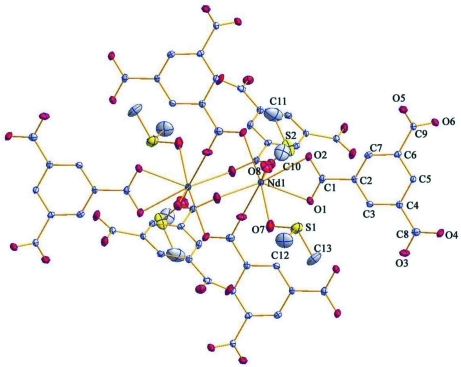
Molecular structure of (I) showing the atom-numbering scheme of the asymmetric unit and symmetry expanded metal-organic framework structure. Displacement ellipsoids are drawn at the 30% probability level.

## supplementary crystallographic information

### Comment

Metal-organic framework design and construction is currently a flourishing field of research owing to the intriguing molecular topologies and the potentially exploitable adsorption (Sun *et al.*, 2006), catalytic (Ravon *et al.*, 2008) and fluorescence (Allendorf *et al.*, 2009) properties of these types of compounds. As functional metal centers, rare earth metals are attracting more attention from synthetic chemists for their unusual coordination properties and special chemical characteristics arising from interactions with the 4f electrons and the propensity to form isostructural complexes (Thirumurugan & Natarajan, 2004). Many coordination polymers utilizing the rare earth elements have been synthesized (Guo *et al.*, 2006).

The title compound poly[(benzene-1,3,5-tricarboxylato)bis(dimethyl sulfoxide)neodymium(III)]n represents a rare-earth three-dimensional metal-organic framework structure (Fig. 1). In this compound, the asymmetric unit, Nd(C_9_H_3_O_6_)(C_2_H_6_OS)_2_, contains one eight-coordinated Nd^3+^ ion, one benzene-1,3,5-tricarboxylate ligand and two coordinated dimethyl sulfoxide molecules, without any guest molecule. Each of two metal center Nd^3+^ ions in a formed cluster is coordinated with six oxygen atoms from four carboxylate groups of the *1,3,5*- benzenetricarboxylic ligands and two oxygen atoms from two terminal dimethyl sulfoxide molecules. Upon symmetry expansion of the asymmetric unit, the metal organic cluster formed therefore consists of two metal centers and four benzene-1,3,5-tricarboxylate groups creating a paddle-wheel type building block arrangement. The remaining coordination sites are occupied by additional benzene-1,3,5-tricarboxylate groups and dimethyl sulfoxide molecules forming a polymeric rare earth three-dimensional metal-organic framework structure.

### Experimental

All reagents were of analytical grade. A mixture of neodymium nitrate (40 mg, 0.10 mmol) and benzene-*1,3,5*-tricarboxylate acid (10 mg, 0.05 mmol) was dissolved in *N,N'*-dimethylformamide (15 ml) and dimethyl sulfoxide (10 ml) at room temperature. Two drops of NaOH (aq, 2 *M*) was added, followed by some nitric acid (aq, 2 *M*) until the solution is clear. This mixture was placed at 55°C for 20 days giving rise to purple rod crystals suitable for *x*-ray crystallographic analysis.

### Refinement

H atoms bound to C atoms were placed in calculated positions and treated as riding on their parent atoms, with C—H = 0.93 Å (aromatic C), C—H = 0.96 Å (methly C) and with *U*_iso_(H) = 1.2Ueq(C). S1 and S2 have been restrained with *DFIX* = 0.02.

### Crystal data

**Table:**

[Nd(C_9_H_3_O_6_)(C_2_H_6_OS)_2_]	*F*(000) = 996
*M**_r_* = 507.61	*D*_x_ = 1.965 Mg m^−^^3^
Monoclinic, *P*2_1_/*n*	Mo *K*α radiation, λ = 0.71073 Å
Hall symbol: -P 2yn	Cell parameters from 2282 reflections
*a* = 10.380 (2) Å	θ = 2.3–24.8°
*b* = 10.752 (3) Å	µ = 3.31 mm^−^^1^
*c* = 16.025 (4) Å	*T* = 273 K
β = 106.419 (4)°	Rod, purple
*V* = 1715.6 (7) Å^3^	0.50 × 0.40 × 0.40 mm
*Z* = 4	

### Data collection

**Table:**

Bruker APEX CCD diffractometer	3008 independent reflections
Radiation source: fine-focus sealed tube	2453 reflections with *I* > 2σ(*I*)
graphite	*R*_int_ = 0.049
Detector resolution: 8.33 pixels mm^-1^	θ_max_ = 25.0°, θ_min_ = 2.1°
phi and ω scans	*h* = −11→12
Absorption correction: multi-scan (*SADABS*; Sheldrick, 2008)	*k* = −10→12
*T*_min_ = 0.289, *T*_max_ = 0.351	*l* = −18→19
8925 measured reflections	

### Refinement

**Table:**

Refinement on *F*^2^	Primary atom site location: structure-invariant direct methods
Least-squares matrix: full	Secondary atom site location: difference Fourier map
*R*[*F*^2^ > 2σ(*F*^2^)] = 0.039	Hydrogen site location: inferred from neighbouring sites
*wR*(*F*^2^) = 0.082	H-atom parameters constrained
*S* = 0.98	*w* = 1/[σ^2^(*F*_o_^2^) + (0.0335*P*)^2^] where *P* = (*F*_o_^2^ + 2*F*_c_^2^)/3
3008 reflections	(Δ/σ)_max_ < 0.001
221 parameters	Δρ_max_ = 1.92 e Å^−^^3^
0 restraints	Δρ_min_ = −0.83 e Å^−^^3^

### Special details

**Table:**

Geometry. All e.s.d.'s (except the e.s.d. in the dihedral angle between two l.s. planes) are estimated using the full covariance matrix. The cell e.s.d.'s are taken into account individually in the estimation of e.s.d.'s in distances, angles and torsion angles; correlations between e.s.d.'s in cell parameters are only used when they are defined by crystal symmetry. An approximate (isotropic) treatment of cell e.s.d.'s is used for estimating e.s.d.'s involving l.s. planes.
Refinement. Refinement of *F*^2^ against ALL reflections. The weighted *R*-factor *wR* and goodness of fit *S* are based on *F*^2^, conventional *R*-factors *R* are based on *F*, with *F* set to zero for negative *F*^2^. The threshold expression of *F*^2^ > σ(*F*^2^) is used only for calculating *R*-factors(gt) *etc*. and is not relevant to the choice of reflections for refinement. *R*-factors based on *F*^2^ are statistically about twice as large as those based on *F*, and *R*- factors based on ALL data will be even larger.

### Fractional atomic coordinates and isotropic or equivalent isotropic displacement parameters (Å^2^)

**Table:**

	*x*	*y*	*z*	*U*_iso_*/*U*_eq_	
Nd1	0.36554 (3)	0.12931 (3)	0.02773 (2)	0.01539 (12)	
S1	0.0680 (2)	0.2768 (2)	−0.08861 (14)	0.0492 (6)	
S2	0.3373 (3)	0.4585 (2)	0.08367 (18)	0.0664 (8)	
O1	0.1446 (4)	0.0876 (4)	0.0571 (3)	0.0280 (11)	
O2	0.2784 (4)	0.2139 (4)	0.1508 (3)	0.0301 (11)	
O3	−0.3430 (4)	0.0586 (4)	0.0532 (3)	0.0297 (11)	
O4	−0.4178 (4)	0.1981 (4)	0.1279 (3)	0.0360 (12)	
O5	0.0801 (4)	0.4650 (4)	0.3619 (3)	0.0268 (11)	
O6	−0.0472 (4)	0.3271 (4)	0.4070 (3)	0.0309 (11)	
O7	0.1768 (5)	0.1920 (5)	−0.1004 (3)	0.0462 (14)	
O8	0.3616 (5)	0.3615 (4)	0.0214 (3)	0.0476 (14)	
C1	0.1662 (6)	0.1639 (5)	0.1191 (4)	0.0204 (15)	
C2	0.0514 (6)	0.1983 (6)	0.1562 (4)	0.0179 (14)	
C3	−0.0760 (6)	0.1517 (5)	0.1195 (4)	0.0196 (14)	
H3	−0.0916	0.0987	0.0719	0.024*	
C4	−0.1821 (6)	0.1837 (6)	0.1535 (4)	0.0190 (14)	
C5	−0.1545 (6)	0.2560 (5)	0.2286 (4)	0.0202 (15)	
H5	−0.2228	0.2735	0.2540	0.024*	
C6	−0.0262 (6)	0.3024 (5)	0.2661 (4)	0.0165 (13)	
C7	0.0737 (6)	0.2764 (5)	0.2272 (4)	0.0196 (14)	
H7	0.1579	0.3123	0.2494	0.024*	
C8	−0.3236 (6)	0.1436 (6)	0.1090 (4)	0.0223 (15)	
C9	0.0043 (6)	0.3722 (6)	0.3517 (4)	0.0204 (14)	
C10	0.3202 (10)	0.6008 (7)	0.0260 (7)	0.080 (3)	
H10A	0.3911	0.6083	−0.0013	0.121*	
H10B	0.3251	0.6687	0.0657	0.121*	
H10C	0.2350	0.6025	−0.0176	0.121*	
C11	0.4971 (10)	0.4797 (10)	0.1533 (7)	0.106 (4)	
H11A	0.5345	0.4005	0.1752	0.159*	
H11B	0.4922	0.5318	0.2010	0.159*	
H11C	0.5531	0.5186	0.1224	0.159*	
C12	0.0497 (10)	0.3915 (8)	−0.1710 (6)	0.075 (3)	
H12A	0.1285	0.4430	−0.1580	0.113*	
H12B	−0.0275	0.4420	−0.1733	0.113*	
H12C	0.0383	0.3515	−0.2262	0.113*	
C13	−0.0810 (8)	0.1947 (9)	−0.1314 (7)	0.079 (3)	
H13A	−0.0857	0.1672	−0.1892	0.119*	
H13B	−0.1561	0.2478	−0.1333	0.119*	
H13C	−0.0832	0.1239	−0.0953	0.119*	

### Atomic displacement parameters (Å^2^)

**Table:**

	*U*^11^	*U*^22^	*U*^33^	*U*^12^	*U*^13^	*U*^23^
Nd1	0.01401 (19)	0.01665 (19)	0.01629 (19)	0.00024 (16)	0.00554 (13)	−0.00076 (15)
S1	0.0391 (12)	0.0561 (14)	0.0477 (14)	0.0094 (11)	0.0047 (10)	0.0119 (10)
S2	0.102 (2)	0.0324 (12)	0.082 (2)	−0.0009 (13)	0.0536 (17)	−0.0043 (12)
O1	0.024 (3)	0.037 (3)	0.027 (3)	−0.002 (2)	0.012 (2)	−0.012 (2)
O2	0.019 (3)	0.037 (3)	0.037 (3)	−0.004 (2)	0.014 (2)	−0.011 (2)
O3	0.032 (3)	0.024 (3)	0.028 (3)	−0.003 (2)	0.001 (2)	−0.009 (2)
O4	0.014 (3)	0.049 (3)	0.045 (3)	−0.001 (2)	0.008 (2)	−0.017 (3)
O5	0.031 (3)	0.028 (3)	0.023 (3)	−0.011 (2)	0.009 (2)	−0.006 (2)
O6	0.033 (3)	0.039 (3)	0.025 (3)	−0.011 (2)	0.015 (2)	−0.013 (2)
O7	0.032 (3)	0.065 (4)	0.045 (4)	0.019 (3)	0.017 (3)	0.012 (3)
O8	0.071 (4)	0.023 (3)	0.053 (4)	−0.001 (3)	0.025 (3)	−0.009 (2)
C1	0.019 (4)	0.023 (4)	0.021 (4)	0.001 (3)	0.010 (3)	0.004 (3)
C2	0.016 (3)	0.024 (4)	0.015 (3)	−0.002 (3)	0.008 (3)	0.000 (3)
C3	0.020 (3)	0.023 (4)	0.015 (3)	0.002 (3)	0.003 (3)	−0.003 (3)
C4	0.017 (3)	0.023 (3)	0.019 (4)	0.000 (3)	0.007 (3)	0.000 (3)
C5	0.014 (3)	0.023 (4)	0.025 (4)	−0.002 (3)	0.009 (3)	−0.005 (3)
C6	0.015 (3)	0.015 (3)	0.018 (4)	0.002 (3)	0.002 (3)	0.001 (3)
C7	0.015 (3)	0.021 (3)	0.021 (4)	−0.001 (3)	0.002 (3)	0.000 (3)
C8	0.025 (4)	0.021 (4)	0.020 (4)	−0.004 (3)	0.004 (3)	0.004 (3)
C9	0.015 (3)	0.028 (4)	0.019 (4)	0.001 (3)	0.007 (3)	−0.006 (3)
C10	0.091 (8)	0.038 (6)	0.118 (10)	0.004 (5)	0.038 (7)	−0.004 (5)
C11	0.112 (10)	0.112 (10)	0.072 (9)	0.054 (8)	−0.009 (7)	−0.003 (7)
C12	0.098 (8)	0.054 (6)	0.065 (7)	0.008 (5)	0.009 (6)	0.035 (5)
C13	0.029 (5)	0.106 (8)	0.092 (9)	−0.007 (5)	−0.001 (5)	−0.004 (7)

### Geometric parameters (Å, °)

**Table:**

Nd1—O3^i^	2.376 (4)	C1—C2	1.519 (8)
Nd1—O6^ii^	2.403 (4)	C2—C3	1.381 (8)
Nd1—O5^iii^	2.451 (4)	C2—C7	1.380 (8)
Nd1—O4^iv^	2.478 (4)	C3—C4	1.401 (8)
Nd1—O7	2.497 (5)	C3—H3	0.9300
Nd1—O8	2.498 (4)	C4—C5	1.394 (8)
Nd1—O1	2.509 (4)	C4—C8	1.503 (8)
Nd1—O2	2.559 (4)	C5—C6	1.390 (8)
Nd1—C1	2.878 (6)	C5—H5	0.9300
S1—O7	1.505 (5)	C6—C7	1.381 (8)
S1—C13	1.744 (8)	C6—C9	1.515 (8)
S1—C12	1.777 (8)	C7—H7	0.9300
S2—O8	1.513 (5)	C10—H10A	0.9600
S2—C11	1.733 (10)	C10—H10B	0.9600
S2—C10	1.770 (9)	C10—H10C	0.9600
O1—C1	1.258 (7)	C11—H11A	0.9600
O2—C1	1.253 (7)	C11—H11B	0.9600
O3—C8	1.255 (7)	C11—H11C	0.9600
O3—Nd1^i^	2.376 (4)	C12—H12A	0.9600
O4—C8	1.249 (7)	C12—H12B	0.9600
O4—Nd1^v^	2.478 (4)	C12—H12C	0.9600
O5—C9	1.253 (7)	C13—H13A	0.9600
O5—Nd1^vi^	2.451 (4)	C13—H13B	0.9600
O6—C9	1.255 (7)	C13—H13C	0.9600
O6—Nd1^vii^	2.403 (4)		
			
O3^i^—Nd1—O6^ii^	74.21 (15)	O1—C1—C2	118.9 (5)
O3^i^—Nd1—O5^iii^	75.39 (15)	O2—C1—Nd1	62.7 (3)
O6^ii^—Nd1—O5^iii^	131.26 (14)	O1—C1—Nd1	60.5 (3)
O3^i^—Nd1—O4^iv^	122.58 (15)	C2—C1—Nd1	170.6 (4)
O6^ii^—Nd1—O4^iv^	89.06 (16)	C3—C2—C7	119.2 (5)
O5^iii^—Nd1—O4^iv^	76.48 (15)	C3—C2—C1	120.5 (5)
O3^i^—Nd1—O7	81.22 (16)	C7—C2—C1	120.3 (5)
O6^ii^—Nd1—O7	70.85 (15)	C2—C3—C4	120.6 (6)
O5^iii^—Nd1—O7	139.05 (16)	C2—C3—H3	119.7
O4^iv^—Nd1—O7	144.05 (17)	C4—C3—H3	119.7
O3^i^—Nd1—O8	146.04 (16)	C5—C4—C3	118.7 (6)
O6^ii^—Nd1—O8	77.17 (15)	C5—C4—C8	120.3 (5)
O5^iii^—Nd1—O8	138.39 (15)	C3—C4—C8	121.0 (5)
O4^iv^—Nd1—O8	74.30 (16)	C6—C5—C4	120.8 (5)
O7—Nd1—O8	72.36 (17)	C6—C5—H5	119.6
O3^i^—Nd1—O1	89.81 (14)	C4—C5—H5	119.6
O6^ii^—Nd1—O1	139.36 (15)	C7—C6—C5	118.8 (5)
O5^iii^—Nd1—O1	76.81 (14)	C7—C6—C9	121.2 (5)
O4^iv^—Nd1—O1	129.95 (14)	C5—C6—C9	119.9 (5)
O7—Nd1—O1	69.90 (15)	C2—C7—C6	121.6 (6)
O8—Nd1—O1	100.41 (15)	C2—C7—H7	119.2
O3^i^—Nd1—O2	136.16 (14)	C6—C7—H7	119.2
O6^ii^—Nd1—O2	147.93 (15)	O4—C8—O3	122.4 (6)
O5^iii^—Nd1—O2	76.07 (14)	O4—C8—C4	118.6 (5)
O4^iv^—Nd1—O2	81.29 (14)	O3—C8—C4	119.1 (6)
O7—Nd1—O2	99.90 (15)	O5—C9—O6	126.3 (6)
O8—Nd1—O2	70.79 (15)	O5—C9—C6	118.4 (5)
O1—Nd1—O2	51.48 (13)	O6—C9—C6	115.3 (5)
O3^i^—Nd1—C1	114.43 (16)	S2—C10—H10A	109.5
O6^ii^—Nd1—C1	150.90 (16)	S2—C10—H10B	109.5
O5^iii^—Nd1—C1	77.15 (15)	H10A—C10—H10B	109.5
O4^iv^—Nd1—C1	106.46 (17)	S2—C10—H10C	109.5
O7—Nd1—C1	82.76 (16)	H10A—C10—H10C	109.5
O8—Nd1—C1	83.44 (16)	H10B—C10—H10C	109.5
O1—Nd1—C1	25.87 (15)	S2—C11—H11A	109.5
O2—Nd1—C1	25.79 (15)	S2—C11—H11B	109.5
O7—S1—C13	104.9 (4)	H11A—C11—H11B	109.5
O7—S1—C12	104.7 (4)	S2—C11—H11C	109.5
C13—S1—C12	99.3 (5)	H11A—C11—H11C	109.5
O8—S2—C11	102.0 (4)	H11B—C11—H11C	109.5
O8—S2—C10	105.2 (4)	S1—C12—H12A	109.5
C11—S2—C10	99.2 (5)	S1—C12—H12B	109.5
C1—O1—Nd1	93.7 (4)	H12A—C12—H12B	109.5
C1—O2—Nd1	91.5 (4)	S1—C12—H12C	109.5
C8—O3—Nd1^i^	168.4 (4)	H12A—C12—H12C	109.5
C8—O4—Nd1^v^	109.4 (4)	H12B—C12—H12C	109.5
C9—O5—Nd1^vi^	132.6 (4)	S1—C13—H13A	109.5
C9—O6—Nd1^vii^	145.7 (4)	S1—C13—H13B	109.5
S1—O7—Nd1	120.2 (3)	H13A—C13—H13B	109.5
S2—O8—Nd1	131.7 (3)	S1—C13—H13C	109.5
O2—C1—O1	122.5 (5)	H13A—C13—H13C	109.5
O2—C1—C2	118.6 (5)	H13B—C13—H13C	109.5
			
O3^i^—Nd1—O1—C1	162.7 (4)	O7—Nd1—C1—O2	−131.4 (4)
O6^ii^—Nd1—O1—C1	−132.2 (4)	O8—Nd1—C1—O2	−58.4 (4)
O5^iii^—Nd1—O1—C1	87.7 (4)	O1—Nd1—C1—O2	170.7 (6)
O4^iv^—Nd1—O1—C1	28.3 (4)	O3^i^—Nd1—C1—O1	−19.0 (4)
O7—Nd1—O1—C1	−116.5 (4)	O6^ii^—Nd1—C1—O1	82.6 (5)
O8—Nd1—O1—C1	−49.8 (4)	O5^iii^—Nd1—C1—O1	−86.2 (4)
O2—Nd1—O1—C1	5.1 (3)	O4^iv^—Nd1—C1—O1	−157.7 (4)
O3^i^—Nd1—O2—C1	−38.5 (4)	O7—Nd1—C1—O1	57.9 (4)
O6^ii^—Nd1—O2—C1	118.6 (4)	O8—Nd1—C1—O1	130.8 (4)
O5^iii^—Nd1—O2—C1	−89.2 (4)	O2—Nd1—C1—O1	−170.7 (6)
O4^iv^—Nd1—O2—C1	−167.4 (4)	O2—C1—C2—C3	175.7 (6)
O7—Nd1—O2—C1	49.1 (4)	O1—C1—C2—C3	−3.3 (9)
O8—Nd1—O2—C1	116.3 (4)	O2—C1—C2—C7	−4.5 (9)
O1—Nd1—O2—C1	−5.2 (3)	O1—C1—C2—C7	176.5 (6)
C13—S1—O7—Nd1	−123.4 (4)	C7—C2—C3—C4	0.4 (9)
C12—S1—O7—Nd1	132.5 (4)	C1—C2—C3—C4	−179.8 (5)
O3^i^—Nd1—O7—S1	144.9 (3)	C2—C3—C4—C5	−4.5 (9)
O6^ii^—Nd1—O7—S1	−138.8 (4)	C2—C3—C4—C8	174.1 (5)
O5^iii^—Nd1—O7—S1	89.5 (4)	C3—C4—C5—C6	4.1 (9)
O4^iv^—Nd1—O7—S1	−79.4 (4)	C8—C4—C5—C6	−174.5 (5)
O8—Nd1—O7—S1	−56.7 (3)	C4—C5—C6—C7	0.5 (9)
O1—Nd1—O7—S1	51.9 (3)	C4—C5—C6—C9	−175.2 (6)
O2—Nd1—O7—S1	9.4 (3)	C3—C2—C7—C6	4.4 (9)
C1—Nd1—O7—S1	28.7 (3)	C1—C2—C7—C6	−175.4 (5)
C11—S2—O8—Nd1	87.3 (5)	C5—C6—C7—C2	−4.9 (9)
C10—S2—O8—Nd1	−169.6 (4)	C9—C6—C7—C2	170.7 (6)
O3^i^—Nd1—O8—S2	157.4 (3)	Nd1^v^—O4—C8—O3	−6.6 (7)
O6^ii^—Nd1—O8—S2	−169.6 (4)	Nd1^v^—O4—C8—C4	171.9 (4)
O5^iii^—Nd1—O8—S2	−29.9 (5)	Nd1^i^—O3—C8—O4	−105 (2)
O4^iv^—Nd1—O8—S2	−76.9 (4)	Nd1^i^—O3—C8—C4	76 (2)
O7—Nd1—O8—S2	116.7 (4)	C5—C4—C8—O4	16.2 (9)
O1—Nd1—O8—S2	51.9 (4)	C3—C4—C8—O4	−162.4 (6)
O2—Nd1—O8—S2	9.2 (4)	C5—C4—C8—O3	−165.3 (6)
C1—Nd1—O8—S2	32.3 (4)	C3—C4—C8—O3	16.1 (9)
Nd1—O2—C1—O1	9.6 (6)	Nd1^vi^—O5—C9—O6	−14.0 (10)
Nd1—O2—C1—C2	−169.4 (5)	Nd1^vi^—O5—C9—C6	168.5 (4)
Nd1—O1—C1—O2	−9.8 (7)	Nd1^vii^—O6—C9—O5	32.1 (11)
Nd1—O1—C1—C2	169.2 (5)	Nd1^vii^—O6—C9—C6	−150.3 (5)
O3^i^—Nd1—C1—O2	151.7 (3)	C7—C6—C9—O5	43.1 (8)
O6^ii^—Nd1—C1—O2	−106.7 (5)	C5—C6—C9—O5	−141.3 (6)
O5^iii^—Nd1—C1—O2	84.5 (4)	C7—C6—C9—O6	−134.6 (6)
O4^iv^—Nd1—C1—O2	13.0 (4)	C5—C6—C9—O6	40.9 (8)

Symmetry codes: (i) −*x*, −*y*, −*z*; (ii) *x*+1/2, −*y*+1/2, *z*−1/2; (iii) −*x*+1/2, *y*−1/2, −*z*+1/2; (iv) *x*+1, *y*, *z*; (v) *x*−1, *y*, *z*; (vi) −*x*+1/2, *y*+1/2, −*z*+1/2; (vii) *x*−1/2, −*y*+1/2, *z*+1/2.
